# Evaluation of Biofilm Formation in *Candida tropicalis* Using a Silicone-Based Platform with Synthetic Urine Medium

**DOI:** 10.3390/microorganisms8050660

**Published:** 2020-05-01

**Authors:** Yi-Kai Tseng, Yu-Chia Chen, Chien-Jui Hou, Fu-Sheng Deng, Shen-Huan Liang, Sin Yong Hoo, Chih-Chieh Hsu, Cai-Ling Ke, Ching-Hsuan Lin

**Affiliations:** 1 Department of Biochemical Science and Technology, College of Life Science, National Taiwan University, Taipei City 100-116, Taiwan; z12345wxyz@gmail.com (Y.-K.T.); r04b22064@ntu.edu.tw (Y.-C.C.); jguitar8177@gmail.com (C.-J.H.); d04b22004@ntu.edu.tw (F.-S.D.); shenhuan0816@gmail.com (S.-H.L.); hoosinyong@hotmail.com (S.Y.H.); niya1711@gmail.com (C.-C.H.); r08b22027@ntu.edu.tw (C.-L.K.); 2 Department of Molecular Microbiology and Immunology, Brown University, Providence, RI 02912, USA

**Keywords:** *Candida tropicalis*, biofilms, synthetic urine, *ROB1*

## Abstract

Molecular mechanisms of biofilm formation in *Candida tropicalis* and current methods for biofilm analyses in this fungal pathogen are limited. (2) Methods: Biofilm biomass and crystal violet staining of the wild-type and each gene mutant strain of *C. tropicalis* were evaluated on silicone under synthetic urine culture conditions. (3) Results: Seven media were tested to compare the effects on biofilm growth with or without silicone. Results showed that biofilm cells of *C. tropicalis* were unable to form firm biofilms on the bottom of 12-well polystyrene plates. However, on a silicone-based platform, Roswell Park Memorial Institute 1640 (RPMI 1640), yeast nitrogen base (YNB) + 1% glucose, and synthetic urine media were able to induce strong biofilm growth. In particular, replacement of Spider medium with synthetic urine in the adherence step and the developmental stage is necessary to gain remarkably increased biofilms. Interestingly, unlike *Candida albicans*, the *C. tropicalis* *ROB1* deletion strain but not the other five biofilm-associated mutants did not cause a significant reduction in biofilm formation, suggesting that the biofilm regulatory circuits of the two species are divergent. (4) Conclusions: This system for *C. tropicalis* biofilm analyses will become a useful tool to unveil the biofilm regulatory network in *C. tropicalis*.

## 1. Introduction

Fungal infections caused by *Candida* species commonly occur in the skin, genitals, or mucous membrane [1]. Lethal candidiasis occurs in immunocompromised patients and is usually in the form of invasive candidiasis [2,3]. In Europe and the United States, the dominant *Candida* species of candidiasis include *Candida albicans* (~50%), *Candida glabrata* (~30%), *Candida parapsilosis* (~12%), *Candida tropicalis* (~7%), and *Candida krusei* (<1%) [4]. However, in tropical and subtropical areas, *C. tropicalis* is wildly found and has become the dominant fungal pathogen in non-*albicans* Candida species [5,6,7,8]. Moreover, *C. tropicalis* develops fluconazole resistance much more rapidly than *C. albicans* [9]. Indeed, the increasing rate of fluconazole resistance in *C. tropicalis* isolates (15%) is greater than that in *C. albicans* isolates (4%) [10,11]. *C. tropicalis* isolated from the environment and soil also exhibits reduced susceptibility to fluconazole due to the usage of agricultural azole drugs [11]. However, compared to *C. albicans*, *C. tropicalis* has been relatively less investigated.

Biofilms are mixed communities of microbes that adhere to a surface and are embedded within an extracellular matrix comprised of polysaccharides, proteins, and extracellular DNA [12]. Biofilms can grow on in-dwelling medical devices and therefore are highly associated with virulence and drug resistance, thereby hampering clinical treatment [13,14]. The formation of mature biofilms in *C. albicans* requires four major distinct steps: early, intermediate, maturation, and dispersal [12]. During the early stage, planktonic cells are present as in the yeast phase and must adhere to a surface. Later, pseudohyphae and hyphae are formed. The increased hyphal growth further promotes extracellular matrix production, leading to biofilm maturation and eventual dispersal [15]. Central to the understanding of the mechanisms underlying *C. albicans* biofilm formation is the regulation by a complex regulatory network in which several major transcriptional factors are involved in biofilm growth [16,17,18]. Here, we focus on six major regulators, Bcr1, Brg1, Efg1, Rob1, Ndt80, and Tec1 [16]. Loss of any one of these regulators in *C. albicans* significantly compromises biofilm formation in vitro and in vivo [16].

In *C. tropicalis*, a flow model has been used to examine *C. tropicalis* biofilm formation on latex and silicone catheters using synthetic urine (SU) medium [19]. Bovine serum- and Spider-induced biomass dry weight to determine *C. tropicalis* biofilms on the bottom of polystyrene plates have also been reported [20]. The former assay requires additional equipment; the latter analysis highly depends on the wash step that may cause variability with different scientists. Hence, to obtain optimal biofilm formation with *C. tropicalis*, several culturing conditions for biofilm development were evaluated in this study. We found that using SU in the adherence step enables *C. tropicalis* to form biofilms on silicone. Moreover, we showed that presence of magnesium in SU is necessary for proliferation and biofilm formation by *C. tropicalis*. Furthermore, deletion of the *BCR1*, *BRG1*, *TEC1*, *EFG1*, or *NDT80* genes but not the *ROB1* gene in *C. tropicalis* reduced biofilm biomass. Reintroduction of the *C. tropicalis ROB1* into *C. albicans rob1Δ* mutant strains could not restore biofilm growth. These results suggest divergent functions of *C. tropicalis ROB1* (*CtROB1*) compared to that of its relative species *C. albicans*. Overall, we established a method for *C. tropicalis* biofilm analysis on silicone using SU medium. This method will help the field study the molecular mechanisms of biofilm formation in *C. tropicalis*.

## 2. Materials and Methods

### 2.1. Media and Reagents

Yeast extract-peptone-dextrose (YPD), Roswell Park Memorial Institute 1640 (RPMI 1640), Lee’s glucose, synthetic defined (SD), and synthetic complete dextrose (SCD) media used in experiments were prepared as described previously [19,21,22,23,24,25]. Notably, mannitol in Spider medium was replaced with 1% glucose for *C. tropicalis* biofilm analyses. SU (pH 5.8) was prepared according to previous reports [19,24]. All chemicals were purchased from Sigma-Aldrich Chemical Co. (St. Louis, MO, USA) unless otherwise stated.

### 2.2. Plasmid and Strain Construction

The yeast strains and primers used in this study are listed in Table 1 and Table S1. All *C. tropicalis* mutants were derived from the *C. tropicalis* MYA3404 strain [26]. To generate the *C. tropicalis tec1Δ*, *bcr1Δ*, *brg1Δ*, *rob1Δ*, *efg1Δ*, and *ndt80Δ* strains, the 5’ and 3’ flanking regions of *TEC1* (primers 505/506 and 507/508), *BCR1* (primers 549/550 and 551/552), *BRG1* (primers 609/610 and 611/612), *ROB1* (primers 625/626 and 627/628), *EFG1* (primers 675/676 and 677/678), and *NDT80* (primers 601/602 and 603/604) were PCR amplified using the indicated primers. The respective 5’ and 3’ PCR products were digested with *Apa*I/*Xho*I and *Sac*II/*Sac*I and cloned into the plasmid pSFS2A [27] to generate the plasmids pSFS-ctTec1 KO, pSFS-ctBcr1 KO, pSFS-ctBrg1 KO, pSFS-ctRob1 KO, pSFS-ctRob1 KO, pSFS-ctEfg1 KO, and pSFS-ctNdt80 KO. Each plasmid was digested with *Apa*I/*Sac*I and transformed into YL477 (MYA3404) [26] to generate heterozygous *Δtec1*/*TEC1*, *Δbcr1*/*BCR1*, *Δbrg1*/*BRG1*, *Δrob1*/*ROB1*, *Δefg1*/*EFG1*, and *Δndt80*/*NDT80* strains. The *SAT1* marker was recycled under treatment with 2% maltose. The heterozygous strains were retransformed with the respective deletion construct to generate the *tec1Δ* (YL1380 and YL1381), *bcr1Δ* (YL1404 and YL1406), *brg1Δ* (YL1402 and YL1403), *rob1Δ* (YL1413 and YL1414), *efg1Δ* (YL1410 and YL1411), and *ndt80Δ* (YL1382 and YL1384) mutant strains. Primers 509/6, 7/510, and 511/512 were used to verify the *tec1Δ* genotype. Primers 553/6, 7/554, and 555/556 were used to verify the *bcr1Δ* genotype. Primers 613/6, 7/614, and 615/616 were used to verify the *brg1Δ* genotype. Primers 629/6, 7/630, and 631/632 were used to verify the *rob1Δ* genotype. Primers 679/6, 7/680, and 681/682 were used to verify the *efg1Δ* genotype. Primers 605/6, 7/606, and 607/608 were used to verify the *ndt80Δ* genotype.

The *TEC1*, *BCR1*, *BRG1*, *ROB1*, *EFG1*, and *NDT80* complementation constructs were made by amplification of their endogenous promoters and *ORF*s using primers 937/938, primers 929/930, primers 931/932, primers 935/936, primers 805/806, and primers 933/934, respectively. The *TEC1*, *BCR1*, *ROB1*, *EFG1*, and *NDT80* PCR products were digested with *Apa*I/*Xho*I and cloned into pSFS2A to generate pSFS-ctTEC1 AB, pSFS-ctBCR1 AB, pSFS-ctROB1 AB, pSFS-ctEFG1 AB, and pSFS-ctNDT80 AB, respectively, whereas the BCR1 PCR product was digested with *Apa*I/*BamH*I and cloned into pSFS2A to generate pSFS-ctBCR1 AB. To generate the complementary strains, plasmid pSFS-ctTEC1 AB was linearized with *Pme*I and transformed into mutants to create *Δtec1*/*Δtec1::TEC1,* YL1498, and YL1499. Plasmid pSFS-ctBCR1 AB was digested with *Bgl*II and transformed into mutants to create *Δbcr1*/*Δbcr1::BCR1,* YL1493, and YL1494. Plasmid pSFS-ctBRG1 AB was partially digested with *Bsa*I and transformed into mutants to create *Δbrg1*/*Δbrg1::BRG1,* YL1495, and YL1496. Plasmid pSFS-ctROB1 AB was digested with *Apa*I/*Xho*I and transformed into mutants to create *Δrob1*/*Δrob1::ROB1,* YL1497, and YL1504. Plasmid pSFS-ctEFG1 AB was digested with *Hpa*I and transformed into mutants to create *Δefg1*/*Δefg1::EFG1,* YL1570, and YL1571. Plasmid pSFS-ctNDT80 AB was partially digested with *Apa*LI and transformed into mutants to create *Δndt80*/*Δndt80::NDT80,* YL1572, and YL1573. To avoid ectopic integration, primers 1795/1796, 1797/1798, 1799/1800, 1801/1802, 1803/1804, and 1805/1806 were used to verify the *C. tropicalis CtTEC1*, *CtBCR1*, *CtBRG1*, *CtROB1*, *CtEFG1*, and *CtNDT80* complementary strains, respectively.

To generate the *C. tropicalis ROB1* (*CtROB1*) open-reading frame fused with the *C. albicans ROB1* promoter (*CaROB1p*) construct, *CtROB1* and *CaROB1p* were amplified with primer pair 753 and 754 and primer pair 755 and 756, respectively. Similarly, *CtBCR1* (primers 763/764) and *CaBCR1p* (primers 765/766) as well as *CtEFG1* (primers 745/746) and *CaEFG1p* (primers 747/748) were amplified with indicated primers, respectively. The PCR products were mixed and amplified again with primers 754/755, 764/765, and 746/747 to create *CaROB1p-CtROB1*, *CaBCR1p-CtBCR1*, and *CaEFG1p-CtEFG1* gene fragments, respectively. The fused *CaROB1p-CtROB1*, *CaBCR1p-CtBCR1*, and *CaEFG1p-CtEFG1* fragments were digested with *Apa*I/*Xho*I, *Apa*I/*BamH*I, and *Apa*I/*Xho*I, respectively, and cloned into pSFS2A to construct pSFS-*CaROB1p-CtROB1*, pSFS-*CaBCR1p-CtBCR1*, and pSFS-*CaEFG1p-CtEFG1*, respectively. The pSFS-*CaROB1p-CtROB1* construct was digested with *Pme*I and transformed into the *C. albicans rob1Δ* to generate *CtROB1* expression strains YL1456 and YL1457 in *C. albicans*. The pSFS-*CaBCR1p-CtBCR1* construct was partially digested with *EcoR*I to generate *CtBCR1* expression strains YL1450 and YL1451 in *C. albicans*. pSFS-*CaEFG1p-CtEFG1* construct was digested with *Hpa*I to generate *CtEFG1* expression strains YL1417 and YL1419 in *C. albicans*. These two independent knockout and knock-in strains exhibited similar phenotypes in our pretest experiments. Only one strain was therefore selected for further analysis in this article. To confirm that each construct was integrated into the right position, primers 1807/766, 1808/756, and 1809/748 were used to verify the *CaBCR1p-CtBCR1*, *CaROB1p-CtROB1*, and *CaEFG1p-CtEFG1*, respectively, in *C. albicans*.

### 2.3. Biofilm Assay and Biofilm Staining

To develop the biofilms on the bottom of polystyrene plates, overnight YPD cultures grown at 30° were used to inoculate polystyrene plates pretreated with bovine serum at an OD_600_ of 0.5. Cells were left to adhere to the plates in Spider at 37° and shaking at 100 rpm for 2 h. Plates were then washed with Phosphate-buffered saline (PBS) three times by pipetting after removed supernatants. Fresh Spider medium was added to each well, and plates were further incubated for 48 h at 37° at 100 rpm [20]. Supernatants were removed, and samples were then photographed. The adhered cells were scratched out of the plastic well and quantified by measuring the OD_600_. Determination of slime index (SI) was based on the previous report with slight modifications [29]. SI was calculated from the biofilm formation values obtained by OD method in relation to the growth culture values measured by OD_600_ prior to washing the wells. SI establishes a relation between biofilm formed and culture growth (SI = (biofilm/growth culture) × 100) [29]. 

Established protocols to measure the dry weight of biofilms in a silicone model of biofilms were performed, with slight modifications [21]. Preweighed 1.5 cm × 1.5 cm sterile silicone squares (Bentec Medical, PR72034-06N, Woodland, CA, USA) were preincubated in bovine serum (Gibco, 1861237; ThermoFisher Scientific Inc., Waltham, MA, USA) for 12 h at 37 °C and 100 rpm in a polystyrene 12-well plastic plate. Serum-treated silicone was washed with 2 mL of PBS. The washed silicone was placed in 2 mL of Spider, Lee’s glucose, RPMI, yeast nitrogen base (YNB) + 1% glucose, SD, SCD + 50% serum, or SU medium for adhesion. Notably, mannitol in Spider medium was replaced with 1% glucose for *C. tropicalis* biofilm analyses. *C. tropicalis* and *C. albicans* cells were grown overnight in YPD medium, and approximately 1 × 10^7^ cells were added on top of each silicone square. The inoculated plate was incubated with gentle agitation (100 rpm) at 37 °C for 4 h for adhesion. Silicone squares were washed with 2 mL of PBS, and incubation continued in 2 mL of fresh Spider medium for 24 h at 37 °C with gentle shaking (100 rpm). Supernatants were removed, and silicone squares were allowed to dry overnight before weighing to determine biofilm mass. Four to five replicate biofilms grown in separate wells were used for each strain.

Crystal violet staining was performed by decanting the silicone, with slight modifications [30]. Cells were stained for 30 min with 2 mL of 0.2% aqueous crystal violet per silicone. Silicone was washed with 2 mL of PBS and destained with 1 mL of 95% ethanol for 30 min. Two hundred microliters of the destain solution was placed in a 96-well plate, and absorbance at 520 nm was read using an ELISA reader (Waltham; Thermo Fisher Scientific Inc., Waltham, MA, USA). The experiment was performed in three experimental replicates.

### 2.4. Statistical Analyses

All statistical analyses were performed with Excel software. Differences were analyzed using a one-tailed or two-tailed Student’s *t*-test with a 95% confidence interval.

## 3. Results

### 3.1. Effects of Different Culture Conditions on Biofilm Formation in the C. tropicalis Wild-Type Strain (MYA3404)

**Figure 1:**
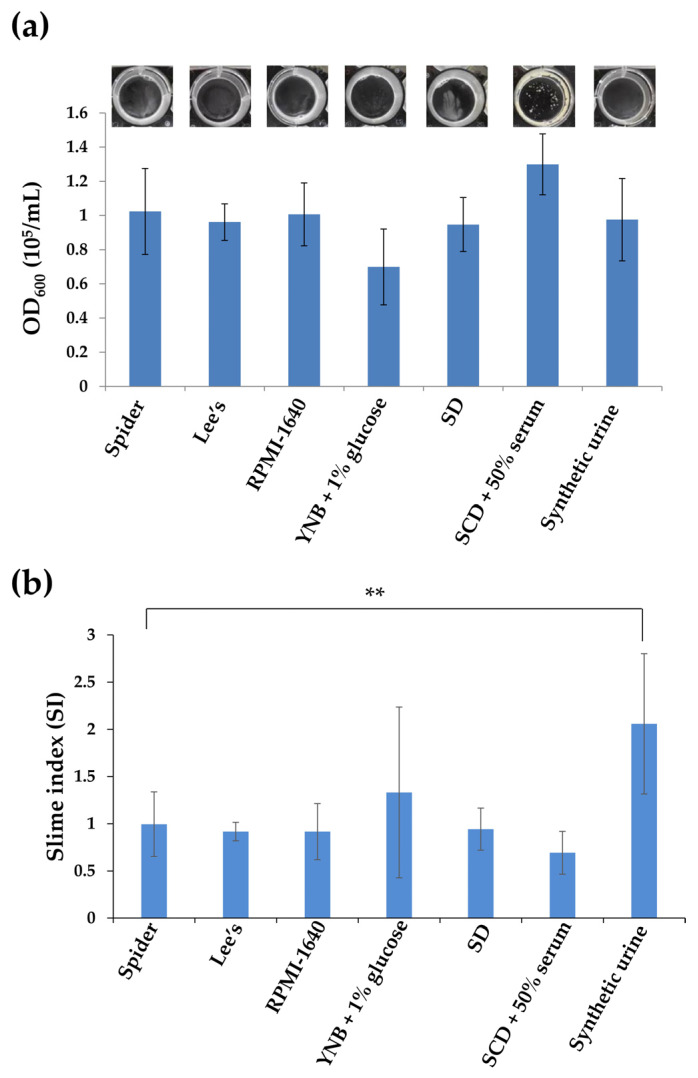
Effects of different culture conditions on *C. tropicalis* biofilm formation on the bottom of 12-well polystyrene plates: (**a**) Representative images of biofilm formation of the *C. tropicalis* wild-type strain (MYA3404) on the bottom of 12-well polystyrene plates. Quantitative results showed that no medium induced cell adhesion and biofilm formation significantly compared to that of the Spider medium on the bottom of 12-well polystyrene plates. Values are the mean ± SD from six replicates. (**b**) *C. tropicalis* exhibited a significant increase in the slime index in synthetic urine medium. Values are the mean ± SD from six replicates. ** *p* < 0.01.

**Figure 2:**
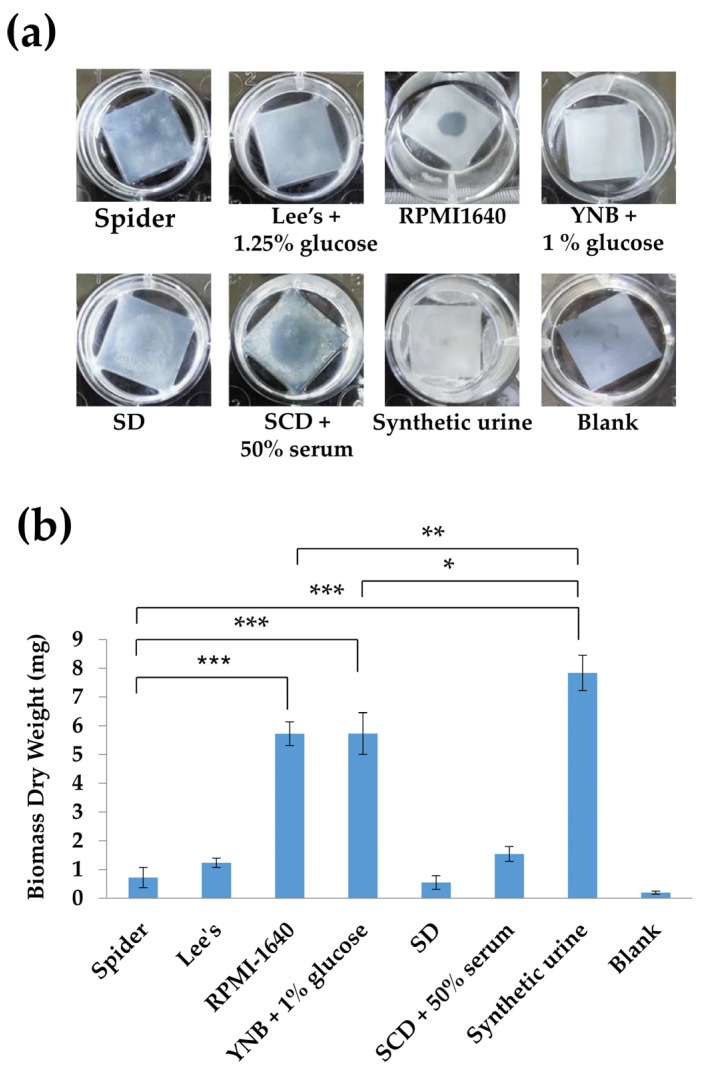
RPMI, YNB + 1% glucose, and synthetic urine (SU) media as adhesion conditions exhibited increased biofilm dry weights. The *C. tropicalis* wild-type strain MYA3404 was cultured under different culture conditions for biofilm analysis in 12-well tissue culture plates with silicone. (**a**) Representative images of biofilm formation of the *C. tropicalis* wild-type strain on silicone. (**b**) Quantitative analysis of the *C. tropicalis* wild-type strain in a biofilm assay on silicone squares: Values are the mean ± SD from five replicates. * *p* < 0.05; ** *p* < 0.01, and *** *p* < 0.001.

We compared the effects of different media during the adhesion step on biofilm growth. These media included Spider, Lee’s glucose, RPMI, YNB + 1% glucose, SD, SCD + 50% serum, and SU media [19,21,22,23,24,25]. We first tested the biofilm cells of *C. tropicalis* on the bottom of 12-well polystyrene plates. However, the biofilm growth of *C. tropicalis* in different culture media was easily washed out, although cells incubated with SCD supplemented with 50% serum for adhesion showed some biofilm formation around the well and a minor amount on the bottom (Figure 1a). We further calculated the slime index (SI), where growth constitutes a correction factor in the determination of biofilm formation. Figure 1b showed that *C. tropicalis* exhibited significant increase in the slime index in SU medium compared to that of the Spider medium. Nevertheless, the final outcome of low quantifiable biofilms by measuring cell density in Figure 1a still caused difficulty in determination. We therefore evaluated whether these culture conditions for the adhesion stage could profoundly affect *C. tropicalis* biofilm formation on silicone-based materials. As shown in Figure 2, the use of RPMI 1640, YNB + 1% glucose, or synthetic urine media as the adherence medium induced the most biofilm growth, particularly with the SU medium. Thus, SU medium was primarily used to analyze *C. tropicalis* biofilm formation on silicone in this work. 

### 3.2. Replacement of Spider Medium with Synthetic Urine in the Adherence Step Profoundly Affected C. tropicalis Biofilm Growth but not that of C. albicans

**Figure 3:**
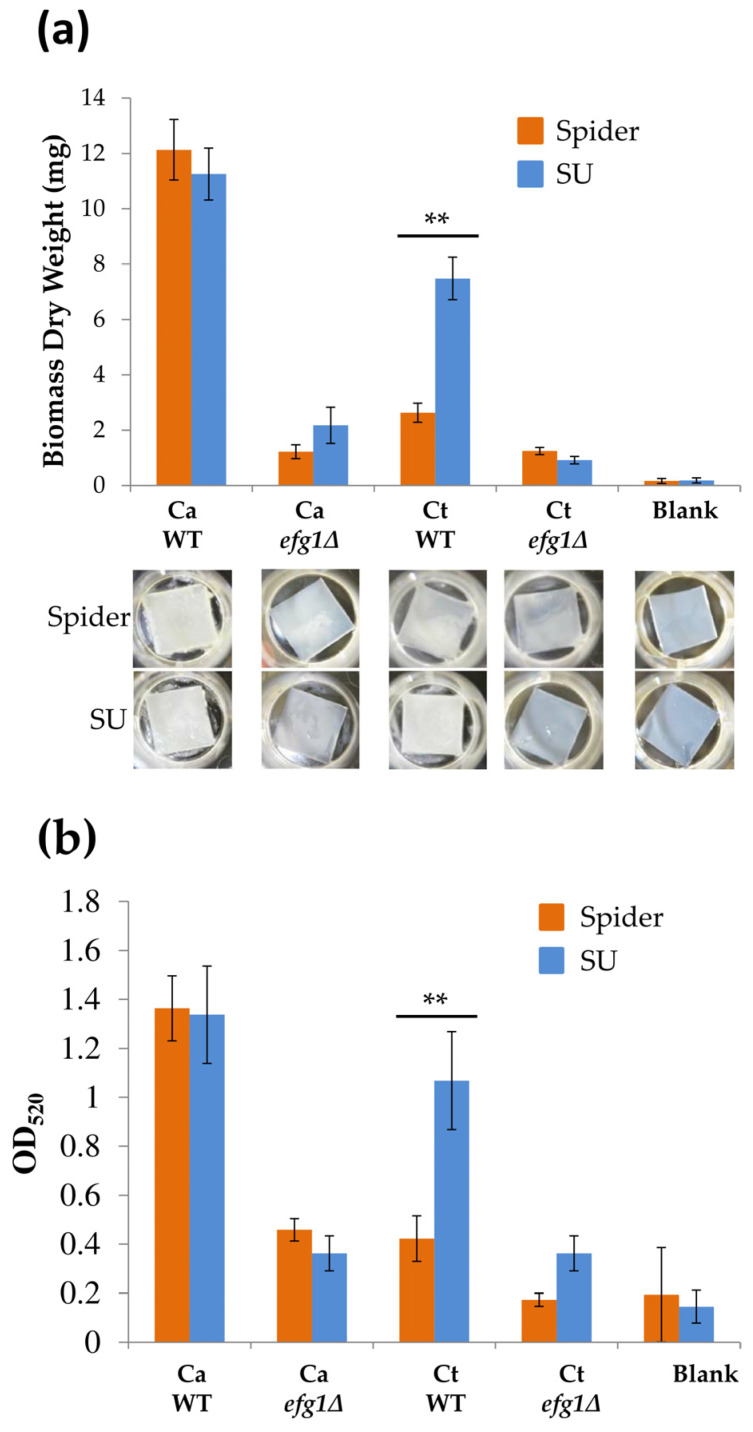
Quantitation of biofilms of *C. albicans* and *C. tropicalis* wild-type (WT) and *efg1Δ* strains in the Spider and SU biofilm formation conditions: (**a**) The use of SU medium resulted in increased *C. tropicalis* biofilm formation, whereas *C. albicans* showed a similar biomass when cultured with Spider medium and SU. Representative images of biofilm formation were shown below. (**b**) The extent of *C. albicans* and *C. tropicalis* biofilms were measured using crystal violet and resulted in similar conclusions in Figure 3a. Values are the mean ± SD from four replicates. ** *p* < 0.01. The *C. albicans efg1Δ* and *C. tropicalis efg1Δ* strains were used as negative controls.

Previous studies have shown that *C. albicans* biofilms were formed in vitro using SU medium [24], and Spider medium has frequently been used for the adhesion step in biofilm development by *C. albicans* [16,21,22]. We therefore evaluated whether changes in the adhesion condition with SU medium affected biofilm development in both *C. albicans* and *C. tropicalis*. The results showed that both Spider and SU media were able to induce biofilm development (~12.1 and ~11.3 mg, respectively) in *C. albicans* (Figure 3a), whereas biomass weights with SU medium significantly increased (~7.7 mg) compared to that with Spider medium (~2.3 mg) in *C. tropicalis* (Figure 3a). Consistent with a previous report, *EFG1* is required for biofilm growth in *C. tropicalis* (Figure 3a), and a mutant was used as a negative control [20]. Biofilm staining using crystal violet also showed similar conclusions, in which more biofilms were stained in SU medium than in Spider medium in the *C. tropicalis* wild-type strain (Figure 3b).

### 3.3. Effects of Depletion of Each Ingredient in SU on C. tropicalis Biofilm Growth

**Figure 4:**
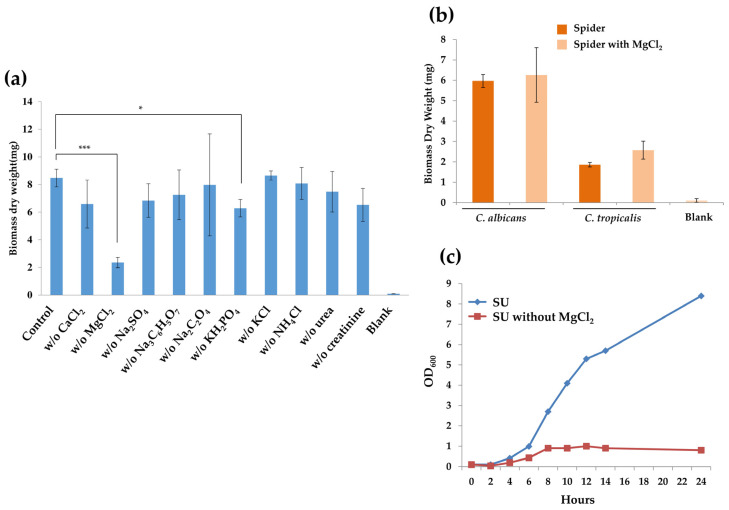
Effects of the ingredients of SU on biofilm formation in *C. tropicalis*: (**a**) Depletion of MgCl_2_ or KH_2_PO_4_ in SU significantly inhibited biofilm formation. (**b**) The addition of exogenous MgCl_2_ in Spider medium caused very only slight effects on biofilm dry weights in both *C. albicans* and *C. tropicalis*. Values are the mean ± SD from five replicates. * *p* < 0.05; *** *p* < 0.001. (**c**) Growth curves of *C. tropicalis* strains at 30 °C in SU with or without additional MgCl_2_ showed the requirement of magnesium for *C. tropicalis* proliferation. Growth rates were monitored every 2 h using a Biowave density meter.

To understand why SU is able to induce *C. tropicalis* biofilm formation, we depleted each ingredient and examined its effect on biofilm growth. The results showed that depletion of MgCl_2_ or KH_2_PO_4_ caused a significant reduction in biofilm formation, especially that of the former (Figure 4a). Lack of CaCl_2_, Na_2_SO_4_, Na_3_C_6_H_5_O_7_, urea, or creatinine resulted in reduced biofilms but without significant effects (Figure 4a). Given that the magnesium chloride concentration in Spider medium (0.25 g/L) is much lower than that in SU medium (0.65 g/L), we further tested whether increases in the magnesium concentration level in Spider medium to the same level as that in SU were able to promote biofilm growth. As shown in Figure 4b, adding exogenous magnesium did not significantly induce biofilm development, suggesting that it could be a combinational effect of SU in biofilm induction. Furthermore, in order to determine whether growth defects occur in SU medium without any magnesium, thereby causing a severe deficiency in biofilm formation, growth curves were analyzed. The results showed that magnesium depletion caused a striking effect on cell growth, indicating that the presence of magnesium is necessary in proliferation, leading to an impact on biofilm formation (Figure 4c).

### 3.4. ROB1 is not Required for Biofilm Development in C. tropicalis

**Figure 5:**
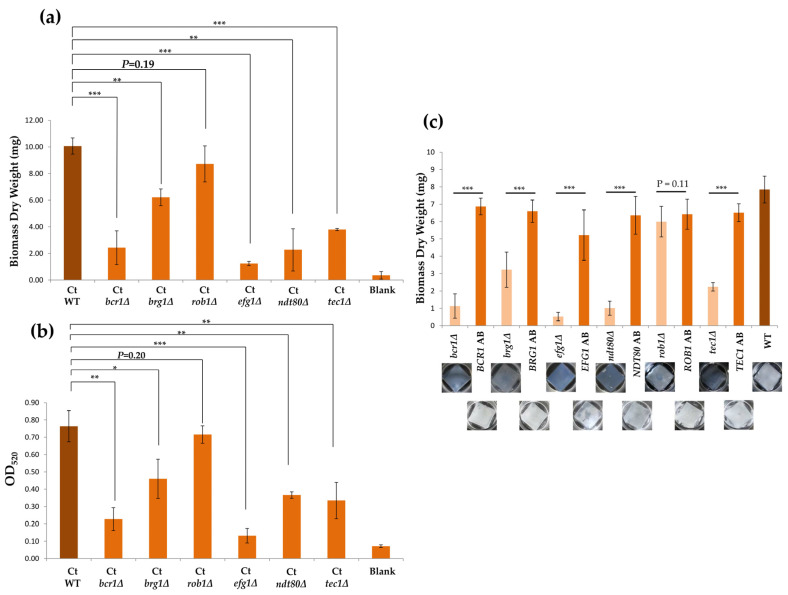
Except the *rob1Δ* mutant strain, the lack of each gene in *C. tropicalis* caused a significant reduction in biofilms. Biofilms were determined by (**a**) biomass dry weight and (**b**) the OD_600_ at the wavelength for crystal violet absorbance. Each experiment was repeated independently at least three times. (**c**) Reintroduction of each functional gene in each respective mutant strain except the *ROB1* complementary strain was able to recover biofilm formation. Values are the mean ± SD from five replicates. * *p* < 0.05, ** *p* < 0.01, and *** *p* < 0.001.

In *C. albicans*, six transcription factors, Bcr1, Brg1, Efg1, Tec1, Rob1, and Ndt80, form a complex regulatory circuit controlling biofilm formation. Loss of each gene causes dramatic biofilm defects [16,21]. Genomic sequence data have shown that the six biofilm-associated genes are also present in *C. tropicalis* [20,26,31]. Six transcription factor amino acid sequences of both *C. albicans* and *C. tropicalis* obtained from the *Candida* Genome Database and UniProt websites were aligned, and alignments were carried out using EMBOSS Pairwise Sequence Alignment. Results showed that *C. tropicalis* Bcr1, Brg1, Efg1, Tec1, Rob1, and Ndt80 share 53.4%, 44%, 55.4%, 61.4%, 35.9%, and 72.1% identities with each respective homolog in *C. albicans* (Table 2). CtBrg1 and CtRob1 exhibited much less identity with CaBrg1 and CaRob1 than the others. To determine whether the homologs of major transcriptional factors play crucial roles in biofilm development in *C. tropicalis*, each *C. tropicalis* homologous transcriptional gene was deleted. Compared with the wild-type strain (MYA3404), five mutant strains of each gene exhibited a significant reduction in biofilm biomasses (Figure 5a). *rob1Δ* showed no effect and no statistically significant difference compared with the wild-type strain. Crystal violet assays also revealed results similar to the previous results on silicone (Figure 5b). Furthermore, reintroduction of a functional gene in each mutant but not *rob1Δ* restored or partially restored biofilm formation in *C. tropicalis* (Figure 5c).

### 3.5. Introduction of the C. tropicalis ROB1 into C. albicans Rob1δ Was Not Able to Restore Biofilm Growth in C. albicans

**Figure 6:**
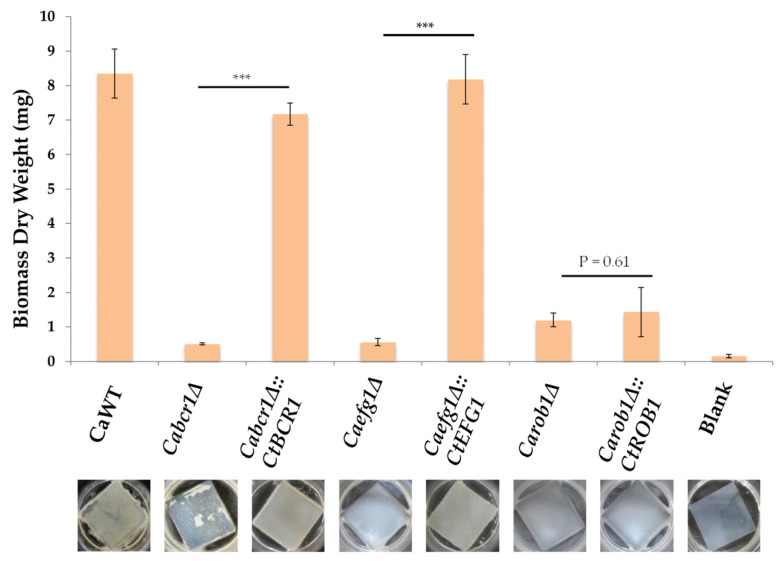
Heterogeneous expression of *C. tropicalis ROB1* in the *C. albicans rob1Δ* could not recover biofilm growth. Values are the mean ± SD from three replicates. Reintroduction of *CtBCR1* and *CtEFG1* into *C. albicans bcr1Δ* and *efg1Δ* strains, respectively, restored biofilm development in *C. albicans*, whereas *Carob1Δ::CtROB1* exhibited few biofilms. *** *p* < 0.001.

To further elucidate the role of *C. tropicalis ROB1* (*CtROB1*) in biofilm formation, we transformed *CtROB1* into the *C. albicans rob1Δ* mutant strains. As shown in Figure 6, *CtROB1* in the *C. albicans rob1Δ* could not promote biofilm development. *CtBCR1* and *CtEFG1* were selected as the parallel control groups, as deletion of any one gene caused severe impairment of biofilm growth in *C. tropicalis* (Figure 5). Results showed that reintroduction of *CtBCR1* and *CtEFG1* into *C. albicans bcr1Δ* and *efg1Δ* strains, respectively, restored biofilm development in *C. albicans* (Figure 6). These findings demonstrated that *CtROB1* does not play an important role in biofilm formation in C. *tropicalis*. Furthermore, the results also suggest that, although *C. tropicalis* and *C. albicans* are evolutionarily closely related, characteristics of biofilm regulatory circuits of the two species are somehow different.

## 4. Discussion

The pathogenicity of chronic and recurrent infections has been associated with the production of biofilms for *C. albicans* and other non-albicans *Candida* species [12,15,22]. It is known that different culture conditions affect biofilm growth in several *Candida* species [19,22,25]. In this study, we established a reliable method for *C. tropicalis* biofilm development with a silicone-based platform. Three media, RPMI 1640, YNB supplemented with 1% glucose, and SU, as an adhesion medium were able to induce strong biofilm growth of *C. tropicalis*. Four media, Spider, Lee’s glucose, SD, and SCD supplemented with 50% serum, produced no or less biofilms. Spider medium contains nutrient broth (beef extract and peptone) to supply amino acids, peptides, and proteins [32]. Similarly, Lee’s glucose and SCD serum media contain a variety of amino acids [22,25,32]. SD medium contains fewer amino acids [22,25,32]. Interestingly, vitamins and inorganic salts are not the major constituents in these media. By contrast, both YNB and RPMI 1640 are rich in vitamins and inorganic salts, although RPMI 1640 also contains a variety of amino acids [22,25,32]. SU medium contains urea, creatinine, and many inorganic salts [19,24]. This fact implies that vitamins and inorganic salts are important for *C. tropicalis* adhesion and biofilm growth on silicone. Moreover, the glucose contents of RPMI 1640 (2 g/L), YNB (9 g/L), and SU (3 g/L) media are lower than those of Spider (10 g/L), Lee’s glucose (12.5 g/L), SD (20 g/L), and SCD + serum (20 g/L) media. High glucose content in a medium may support and promote planktonic growth rather than biofilm development in *C. tropicalis*. Interestingly, the compositions of SU are relatively simple, but this medium induced *C. tropicalis* biofilm formation the most. This result might explain why *Candida* species favor urinary tract infection [33].

After analysis of the effect of each ingredient in SU on biofilm growth, we found that depletion of magnesium exhibited the greatest impact on cell proliferation, thereby affecting biofilm formation. Metal ions are important elements for prokaryotic and eukaryotic cells and play crucial roles in numerous enzymatic reactions to maintain physiological function, including DNA replication, transcription, and biosynthesis of precursors [34,35], but they can also be toxic to living organisms if present in excess [36]. Furthermore, several reports have shown that cation concentrations influence extracellular product formation and biofilm-associated growth in microorganisms. For example, calcium and magnesium cations can enhance *Pseudomonas aeruginosa* adherence ability [37]. Increases in Mg^2+^ concentration promote cell attachment and biofilm formation in both *Staphylococcus aureus* and *Pseudomonas fluorescens* [38,39]. Calcium increases the amount of extracellular matrix composition associated with *Pseudoalteromonas* sp. biofilms [40]. However, 50 mM and higher Mg^2+^ concentrations significantly inhibit biofilm formation in *Bacillus* species [41]. The adherence ability of *Staphylococcus epidermidis* is enhanced in low concentrations of magnesium [42]. In *C. parapsilosis*, an increase in Mn^2+^, with a maximum at 2 mM, significantly induced biofilm formation [43]. Moreover, different metal ions can suppress or enhance *C. albicans* and *C. tropicalis* biofilm formation. For example, Co^2+^, Cu^2+^, Ag^+^, Cd^2+^, Hg^2+^, Pb^2+^, AsO_2_^−^, and SeO_3_^2−^ inhibit hyphal formation in biofilms of both *Candida* species, whereas CrO_4_^2−^ triggers a transition to the hyphal cell morphotype in *C. tropicalis* biofilms [44]. These data indicate that the effects of metal ions on microorganisms are highly dependent on the different types of added metal ions and microbial species.

Spider medium, containing fewer magnesium ions than SU medium, is often used for *C. albicans* biofilm assays, but it fails to induce *C. tropicalis* adhesion and biofilm formation. The addition of exogenous magnesium to the Spider medium resulted in only very slight, nonsignificant increases in biomass dry weights. These results indicated that *C. albicans* and *C. tropicalis* displayed strikingly variable heterogeneous growth, adhesion, and biofilm-forming potential in different growth media. However, the mechanisms of why SU is able to induce *C. albicans* and *C. tropicalis* biofilm formation remain unknown. It is possible that the outcome results from the fact that a number of metal ions, urea, and creatinine have minor effects on biofilm formation, contributing to the impact.

Mechanisms underlying biofilm formation in *C. albicans* are controlled by a central regulatory network with six transcriptional factors: Bcr1, Brg1, Efg1, Tec1, Rob1, and Ndt80 [16]. However, studies regarding biofilm formation mechanisms are very limited in non-albicans *Candida* species, and biofilm formation regulated by the circuit of six transcription factors may vary in *Candida* species. For example, in *C. parapsilosis*, only *EFG1* and *BCR1* deletion strains exhibited similar effects on biofilm formation as those observed in *C. albicans*, whereas *BRG1* and *TEC1* do not play critical roles in biofilm growth in *C. parapsilosis* [45]. Furthermore, in contrast to in *C. albicans*, four transcription factor genes, *CPH2*, *UME6*, *CZF1,* and *GZF3*, were characterized for their roles in biofilm formation and are unique to *C. parapsilosis* [45]. These six homologous genes have also been identified in *C. tropicalis* [20,26]. Interestingly, the degree of sequence identity of two species corresponds to biofilm results, in which the lower identity causes less effect in biofilm formation in *C. tropicalis* mutant strains. Both *C. tropicalis brg1Δ* and *rob1Δ* strains formed more biofilms compared to the other mutant strains, although *brg1Δ* produced reduced biofilm significantly. Furthermore, expression of *CtROB1* in the *C. albicans rob1Δ* was not able to recover biofilms. These findings suggest that, although the functions of five biofilm-associated regulators in both *C. albicans* and *C. tropicalis* are relatively conserved, mechanisms regarding biofilm formation in *C. tropicalis* are distinct from those of its relative *C. albicans*. Furthermore, it will be worthwhile to explore the transcriptional expression profile between planktonic cells and biofilm cells using an SU-induced biofilm platform to understand the *C. tropicalis* biofilm regulatory network.

## Figures and Tables

**Table 1:** Strains used in this study.

Strain	Species	Genotype	Source
SC5314	*C. albicans*	Wild-Type	[26]
RBY717	*C. albicans*	*ura3::imm434*/*URA3* *iro1::imm434*/*IRO1*	[28]
YL477	*C. tropicalis*	wild-type sequence strain (MYA3404)	[26]
YL1344	*C. albicans*	*bcr1* *Δ/bcr1* *Δ*	[21]
YL1346	*C. albicans*	*brg1* *Δ/brg1* *Δ*	[21]
YL1348	*C. albicans*	*efg1* *Δ/efg1* *Δ*	[21]
YL1350	*C. albicans*	*ndt80* *Δ/ndt80* *Δ*	[21]
YL1354	*C. albicans*	*rob1* *Δ/rob1* *Δ*	[21]
YL1356	*C. albicans*	*tec1* *Δ/tec1* *Δ*	[21]
YL1380	*C. tropicalis*	*tec1* *Δ/tec1* *Δ/-SAT1*	This study
YL1381	*C. tropicalis*	*tec1* *Δ/tec1* *Δ/-SAT1*	This study
YL1382	*C. tropicalis*	*ndt80* *Δ/ndt80* *Δ/-SAT1*	This study
YL1384	*C. tropicalis*	*ndt80* *Δ/ndt80* *Δ/-SAT1*	This study
YL1402	*C. tropicalis*	*brg1* *Δ/brg1* *Δ/-SAT1*	This study
YL1403	*C. tropicalis*	*brg1* *Δ/brg1* *Δ/-SAT1*	This study
YL1404	*C. tropicalis*	*bcr1* *Δ/bcr1* *Δ/-SAT1*	This study
YL1406	*C. tropicalis*	*bcr1* *Δ/bcr1* *Δ/-SAT1*	This study
YL1410	*C. tropicalis*	*efg1* *Δ/efg1/-SAT1*	This study
YL1411	*C. tropicalis*	*efg1* *Δ/efg1/-SAT1*	This study
YL1413	*C. tropicalis*	*rob1* *Δ/rob1* *Δ/-SAT1*	This study
YL1414	*C. tropicalis*	*rob1* *Δ/rob1* *Δ/-SAT11*	This study
YL1417	*C. albicans*	*efg1* *Δ/* *efg1* *Δ/::Ct* *EFG* *1-SAT1*	This study
YL1419	*C. albicans*	*efg1* *Δ/* *efg1* *Δ/::Ct* *EFG* *1-SAT1*	This study
YL1450	*C. albicans*	*bcr1* *Δ/* *bcr1* *Δ/::Ct* *BCR* *1-SAT1*	This study
YL1451	*C. albicans*	*bcr1* *Δ/* *bcr1* *Δ/::Ct* *BCR* *1-SAT1*	This study
YL1456	*C. albicans*	*rob1* *Δ/rob1* *Δ/::CtROB1-SAT1*	This study
YL1457	*C. albicans*	*rob1* *Δ/rob1* *Δ/::CtROB1-SAT1*	This study
YL1493	*C. tropicalis*	*bcr1* *Δ/bcr1* *Δ/::BCR1-SAT1*	This study
YL1494	*C. tropicalis*	*bcr1* *Δ/bcr1* *Δ/::BCR1-SAT1*	This study
YL1495	*C. tropicalis*	*brg1* *Δ/brg1* *Δ/::BRG1-SAT1*	This study
YL1496	*C. tropicalis*	*brg1* *Δ/brg1* *Δ/::BRG1-SAT1*	This study
YL1497	*C. tropicalis*	*rob1* *Δ/rob1* *Δ/::ROB1-SAT1*	This study
YL1498	*C. tropicalis*	*tec1* *Δ/tec1* *Δ/::TEC1-SAT1*	This study
YL1499	*C. tropicalis*	*tec1* *Δ tec1* *Δ/::TEC1-SAT1*	This study
YL1504	*C. tropicalis*	*rob1* *Δ rob1* *Δ/::ROB1-SAT1*	This study
YL1570	*C. tropicalis*	*efg1* *Δ/efg1* *Δ/::EFG1-SAT1*	This study
YL1571	*C. tropicalis*	*efg1* *Δ/efg1* *Δ/::EFG1-SAT1*	This study
YL1572	*C. tropicalis*	*ndt80* *Δ/ndt80* *Δ/::NDT80-SAT1*	This study
YL1573	*C. tropicalis*	*ndt80* *Δ/ndt80* *Δ/::NDT80-SAT1*	This study

**Table 2:** Sequence alignment of *C. tropicalis* Bcr1, Brg1, Efg1, Tec1, Rob1, and Ndt80 with the respective homolog in *C. albicans*.

Protein in *C. tropicalis*	Bcr1	Brg1	Efg1	Tec1	Rob1	Ndt80
Identity to respective homolog in *C. albicans*	53.4%	44.0%	55.4%	61.4%	35.9%	72.1%
Similarity to respective homolog in *C. albicans*	62.1%	50.5%	60.2%	73.7%	50%	75.2%

## Supplementary Materials

The following are available online at https://www.mdpi.com/2076-2607/8/5/660/s1, Table S1: Oligonucleotides used in this study.
