# Supplementary material for: Evaluation of Biofilm Formation in Candida tropicalis Using a Silicone-Based Platform with Synthetic Urine Medium
Source: Microorganisms. 2020 May 1;8(5):660. doi: 10.3390/microorganisms8050660 (PMC7284471; doi:10.3390/microorganisms8050660)
Supplement: Supplementary file 1 [file microorganisms-08-00660-s001.pdf]

Table S1. Oligonucleotides used in this study.

Name	Sequence(5′->3′)
6	CTCAACCATAGCAATCATGG
7	GCGAAAAAGTGGGCACTAAG
505	GGAGCGGGGCCCCCCATTGCTTTCTTTTTGGTTTTC
506	GGAGCGCTCGAGTTCTCATTGGAGTAGAAGCAGGTG
507	GGAGCGCCGCGGTACCTTAACTTCTTCTCCCTCCC
508	GGAGCGGAGCTCGGAGTACATAGCCCAAGACCAG
509	CTAAATAAGTGAACTACCCCCCA
510	CCAATTGGAACAATGGTAGTAGC
511	CAAGATCGTGATTTCTTGACTAGAT
512	CGGCAAATTGTTGTTGTTGTTG
549	GGAGCGGGGCCCCCGACAAACACGTCTAATCAATC
550	GGAGCGCTCGAGTGAATGGGAGAAGAAAGGATGTC
551	GGAGCGCCGCGGTGGAGGTGTTGTTTCCCTTTGT
552	GGAGCGGAGCTCTGCCAGGCGAATTAACTTACG
553	TCTGCTGCTGTTGTTGTTGTTG
554	TTTATTGAATGACCTCCTCTCCC
555	CCTCCTCCACCAAAGATTTCTG
556	CATCGTTCAATACTCCTCTAAGCC
601	GGAGCGGGGCCCGCTGCTGCTGCTGCTAATGT
602	GGAGCGCTCGAGCAAATGATCTTCATGGTGTAGTTG
603	GGAGCGCCGCGGGTAAGATACACACAGCAACAGCCAT
604	GGAGCGGAGCTCATACACACAGATTGACTTGCGG
605	CCTGTATCGTTTATATACCACCAGTG
606	CACAGCAGACACAACGAAGGG
607	GGTTAATGGATTCCCACCTCAT
608	GGTGGTGATTCAACAGAACTCAA
609	GGAGCGGGGCCCTGAGTGTGATGACGATGAAGG
610	GGAGCGCTCGAGTAAGTTGTAATGAAATGAAAGCAAC
611	GGAGCGCCGCGGGCATTGGGGGATGGTTCATT
612	GGAGCGGAGCTCTTGATCTTGAAAGTCTGGATTATTC
613	GAGAATGTAGGAAGTGTTGCATC
614	CAGAACAAATTGCGTTTCATTGC
615	CCAAGGTGCTACTGCTGCTG
616	AGGTGGTGGTAATGCAATTCC
625	GGAGCGGGGCCCCCAAGATGGCTGTTGATAAAGTTC

626	GGAGCGCTCGAGGATCTTGATTGATGTTGATCACTTG
627	GGAGCGCCGCGGCACTGAAACTTAGTAGGCCGG
628	GGAGCGGAGCTCCTTGAGAGAAAAGGGTTTGG
629	CATGTCCTGCTTCATTCATTTTG
630	ACGGGTGTCAAGGTGTGGTG
631	CCATACAAGGACGCAGAGGATT
632	CCTCGCTGGTTGACAATTCTT
675	GGAGCGGGGCCCCCACACTGACAGAGATATCC
676	GGAGCGCTCGAGCAAACCTTCTTCTTTTAATGC
677	GGAGCGCCGCGGGGGGAAATTAAATATAGACTACA
678	GGAGCGGAGCTCTAAAGCACTAGGGTGAAGTCC
679	CCCCATCCATTGGAAGTAGATA
680	ACAGAAATATCAACTCAAAGGT
681	AATTATACTAGTGCTCCTGCAG
682	TTTAGTATTACTGGCAGTGGTG
745	ATGTCAACTTATTCCATACCTTATTATAAT
746	GGAGCGCTCGAGTCTATTTTTCTTCTTTCACACCACTATTGT
753	ATGGCAAGTAGCACTTCAACTCGATATCCT
754	GGAGCGCTCGAGTGATACAAAAGATTCCTATAAACCAAATCC
755	GGAGCGGGGCCCGGCCATTTAAGCAGCAGCTTAT
756	AGGATATCGAGTTGAAGTGCTACTTGCCATGGTGTTCCTGAATGT
763	ATGTCAAGTAATACTCAGGTATATCAGAAA
764	GGAGCGGGATCCAATGTATTAAATATTTCATTCTTCTTTGAT
765	GGAGCGGGGCCCTATATATATAAACCAACCAACATTCCAATT
766	TTTCTGATATACCTGAGTATTACTTGACATGATATATATCGAGAT
747	GGAGCGGGGCCCGTAATTTCAATCTTATTTAACTATCCAATT
748	ATTATAATAAGGTATGGAATAAGTTGACATTAATATGGGTTATAT
805	GGAGCGGGGCCCAATGATTGCGATAGAAAGAG
806	GGAGCGCTCGAGCTATTTTTCTTCTTTCACACC
929	GGAGCGGGGCCCACAACAACGATCACAAG
930	GGAGCGGGATCCTCATTCTTCTTTGATCGAAA
931	GGAGCGGGGCCCCATAGTGATATTTTTCCGCA
932	GGAGCGCTCGAGATCATACCATTGTTAAAATG
933	GGAGCGGGGCCCAGAAAGAGGAGCGGAGCATA
934	GGAGCGCTCGAGTTACTGAGGAGTTGGTGTTG
935	GGAGCGGGGCCCCCCTTTGTATTTCTCTTTCC
936	GGAGCGCTCGAGCCTATAAACCAAATCCAGTAG

937	GGAGCGGGGCCCGGTACCACAGGCTATTTATCAC
938	GGAGCGCTCGAGATTAAAATTCGCCAGCAAATC
1795	AGATGGCGTGTCCAAAGCTCAA
1796	AAACAGATGTACATATTACTACG
1797	CCCACTTTAGTCCCACTCGCTT
1798	ATTCAATGGAAATCTGTGAGAC
1799	TGAGTTCCTCGGTACTTCCTTGT
1800	CCTAGTAGACGGATTTTACGAGCACG
1801	TATGCCACGGCGATACACTGAGA
1802	CGACATAGGTTACATCTGAAGTG
1803	GAAGGAAGAATGCACTGTAGGAT
1804	GTGTCCTTTCTTTGAAACCAAAAC
1805	TGCATACTGTACATTATGTAACG
1806	GAAATAATATATGGTGTGAGTGT
1807	AAGTGCGAGAGAGAGAACGACAG
1808	CATCCAATTCAAGTAGTGGCAA
1809	GAATAATCTATCATCTATCAAATAG
